# A mutation in the AdhE alcohol dehydrogenase of *Clostridium thermocellum* increases tolerance to several primary alcohols, including isobutanol, n-butanol and ethanol

**DOI:** 10.1038/s41598-018-37979-5

**Published:** 2019-02-11

**Authors:** Liang Tian, Nicholas D. Cervenka, Aidan M. Low, Daniel G. Olson, Lee R. Lynd

**Affiliations:** 1 Thayer School of Engineering, Dartmouth College, Hanover, NH 03755 USA; 2 Department of Biological Sciences, Dartmouth College, Hanover, NH 03755 USA; 3 Dartmouth College, Hanover, NH 03755 USA; 4 Center for Bioenergy Innovation, Oak Ridge National Laboratory, Oak Ridge, TN 37830 USA

## Abstract

*Clostridium thermocellum* is a good candidate organism for producing cellulosic biofuels due to its native ability to ferment cellulose, however its maximum biofuel titer is limited by tolerance. Wild type *C*. *thermocellum* is inhibited by 5 g/L n-butanol. Using growth adaptation in a chemostat, we increased n-butanol tolerance to 15 g/L. We discovered that several tolerant strains had acquired a D494G mutation in the *adhE* gene. Re-introducing this mutation recapitulated the n-butanol tolerance phenotype. In addition, it increased tolerance to several other primary alcohols including isobutanol and ethanol. To confirm that *adhE* is the cause of inhibition by primary alcohols, we showed that deleting *adhE* also increases tolerance to several primary alcohols.

## Introduction

Metabolic engineering has been widely applied to different hosts for the production of pharmaceuticals, biofuels and bulk chemicals. Producing chemicals at high titer often results in inhibition of the host organism. Therefore, improving tolerance is an essential step in engineering microorganisms to maximize their productivity and develop economically feasible processes^1–3^. Previous studies looking at strategies that microbes use to increase their inhibitor tolerance have focused on changes in the cell membrane, including: lipid composition, membrane fluidity and changes to specific efflux pumps^4–9^. Other strategies include upregulation of chaperones to increase protein stability^10–12^ and mutations in transcription factors that regulate the cellular response to environmental stress^13–15^.

*Clostridium thermocellum* is a good candidate organism for the production of biofuels by consolidated bioprocessing due to its ability to rapidly ferment cellulosic biomass^16,17^. It can natively produce ethanol, and some strains have been engineered to produce ethanol at a titer of 25–30 g/L^18,19^. Currently, tolerance appears to be the main cause of the titer limitation^19,20^. Besides ethanol, *C*. *thermocellum* has the potential to produce a variety of other products, including lactate^21^, amino acids^19,22^ and several advanced biofuel and chemical products^23^. Recently, a heterologous pathway was introduced into *C*. *thermocellum* to increase its isobutanol production^24^.

Historically, n-butanol  has been produced by fermentations involving the related organism, *Clostridium acetobutylicum*^25^, and for this reason, we think n-butanol may be a good candidate biofuel molecule for production in *C*. *thermocellum*. In this work, we applied adaptive laboratory evolution to isolate a strain of *C*. *thermocellum* tolerant to increased concentrations of n-butanol. To understand the genotype-phenotype relationship of this mutant, we re-sequenced the genomes of tolerant mutants, and re-introduced observed mutations to recapitulate the n-butanol tolerance phenotype.

## Results and Discussion

### Isolation and characterization of a strain with improved n-butanol tolerance

**Figure 1:**
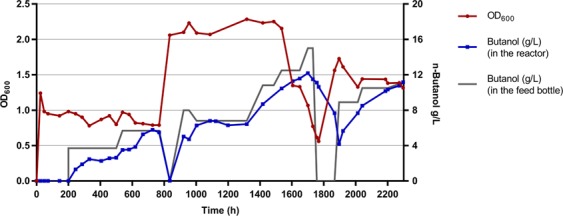
Chemostat culture of the *C*. *thermocellum* for selection of improved n-butanol tolerance. The strains were grown on MTC-5 minimal medium in a bioreactor with pH regulation at 55 °C. From time 0 to 800 hours and 1700 to 2300 hours, the concentration of cellobiose in the feed was 5 g/L . From 800 to 1700 hours, the concentration of cellobiose in the feed was 10 g/L. The red line represents the biomass concentration (as measured by OD_600_, using standard absorbance units). The blue line represents the n-butanol concentration in the bioreactor and the grey line represents the n-butanol concentration in the feed bottle.

First, we tested the tolerance to n-butanol in batch culture and found that 4 g/L inhibited but did not completely eliminate growth (Figure S1). Adaptive laboratory evolution experiments for n-butanol tolerance were carried out in chemostat bioreactors. After more than 2000 hours, butanol tolerance had improved to 10 g/L (Fig. 1), and we purified 12 isolates.

**Figure 2:**
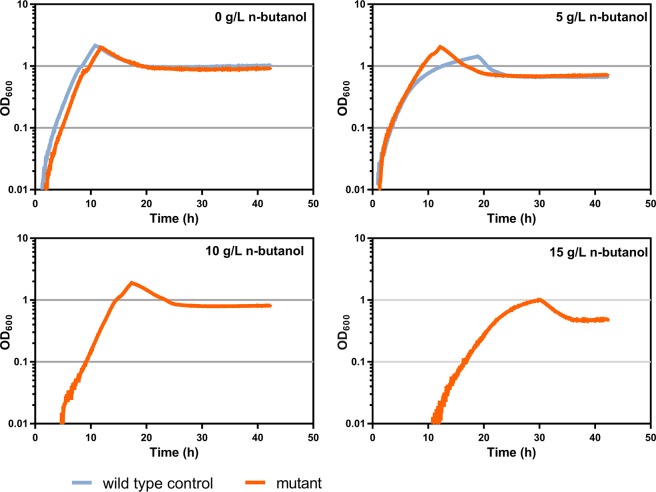
Improved n-butanol tolerance of *C*. *thermocellum* mutants. Growth rate was measured in MTC-5 medium at 55 °C. Growth comparison of wild type and butanol-tolerant mutant in different concentrations of n-butanol. Cells were grown in a 96-well plate and absorbance at 600 nm was measured at 3-minute intervals. Of the 12 isolates selected, all showed similar improvements in n-butanol tolerance, and the results from a single representative isolate are presented. The OD_600_ values are plotted on a semi-logarithmic scale. The orange line represents the OD_600_ value of the mutant strain and the light blue line represents the OD_600_ value of wild type strain. The wild type strain did not grow at n-butanol concentrations above 5 g/L.

To evaluate the n-butanol tolerance of the 12 isolates, we tested the growth of the isolates and the wild type strain in the presence of 0, 5, 10, and 15 g/L n-butanol. All 12 isolates showed increased growth compared with the wild type control with 5 g/L n-butanol, while maintaining similar growth in the absence of n-butanol (Fig. 2). The wild type strain did not grow in the presence of 10 and 15 g/L n-butanol; however, all of the selected isolates were still able to grow. The maximum OD_600_ was slightly lower for 10 g/L n-butanol condition and 50% lower with 15 g/L n-butanol condition, compared to the no-butanol control, and the lag phase was about 2 and 15 hours longer for 10 g/L n-butanol and 15 g/L n-butanol respectively (Fig. 2). These results indicate that evolutionary selection by chemostat was successful in isolating n-butanol tolerant strains of *C*. *thermocellum*.

### Whole-genome sequencing of n-butanol tolerant strains and identification of key mutations

To identify specific mutations in the genome of the n-butanol tolerant strains, we re-sequenced the genomes of 6 isolates. Common mutations among these 6 isolates are listed in Table 1.

**Table 1:** Common mutations among the 6 selected n-butanol tolerant strains.

Region^a^	*C*. *thermocellum* DSM 1313 genome (CDS)	Type	Ref	Allele	Gene product
309131	Clo1313_0284 upstream	SNV	G	A	hypothetical protein
493829^493830	Clo1313_0438	Insertion	—	T	transcription elongation factor GreA
590805^590806	Clo1313_0538 upstream	Insertion	—	C	hypothetical protein
904318	Clo1313_0785	SNV	G	T	transposase mutator type
989039^989040	Clo1313_0853	Insertion	—	A	phospholipase D/Transphosphatidylase
2097217	Clo1313_1798	SNV	T	C	iron-containing alcohol dehydrogenase

^a^Genome coordinates based on NC_017304.1 sequence from Genbank.

Among the 6 common mutations, the mutations in coding sequences of *Clo1313_0853* and *Clo1313_1798* were found in a previous study looking at ethanol tolerance in *C*. *thermocellum*^26^. The gene *Clo1313_0853* is annotated to encode a phospholipase D enzyme (PLD), which catalyzes the hydrolysis of phosphatidylcholine and other phospholipids to generate phosphatidic acid (PA), a necessary structural element of membranes. However, in the presence of a primary alcohol, PLD activity can generate phosphatidyl alcohol instead of PA^27,28^, and the resulting PA deficiency may be toxic. The mutation in the *Clo1313_0853* gene truncates the protein by frameshift, which should eliminate activity. This mutation could protect the membrane when ethanol or n-butanol is present. The mutation in the *Clo1313_1798* gene, the bifunctional alcohol dehydrogenase (*adhE*) generates a D494G amino acid change. This mutation has been previously shown to increase the ability of the alcohol dehydrogenase reaction to use NADPH as a cofactor^29^. It has also been shown to increase ethanol yield and titer^30^, however its effect on ethanol tolerance has not been studied.

### Reconstruction of n-butanol tolerance

To determine whether mutations in *Clo1313_0853* and *Clo1313_1798* were the cause of the observed increase in n-butanol tolerance, we set out to reconstruct the phenotype by introducing specific mutations in the wild type strain. For *Clo1313_0853*, we inactivated the gene by deleting it (strain LL1636, Table 3). This strain did not show any obvious improvement in n-butanol or ethanol tolerance compared to the control. For the *adhE*^*D494G*^ mutation, we have a pair of strains that differ only by its absence or presence (strains LL1160 and LL1161 respectively, Table 3), whose construction is described elsewhere^31^.

**Figure 3:**
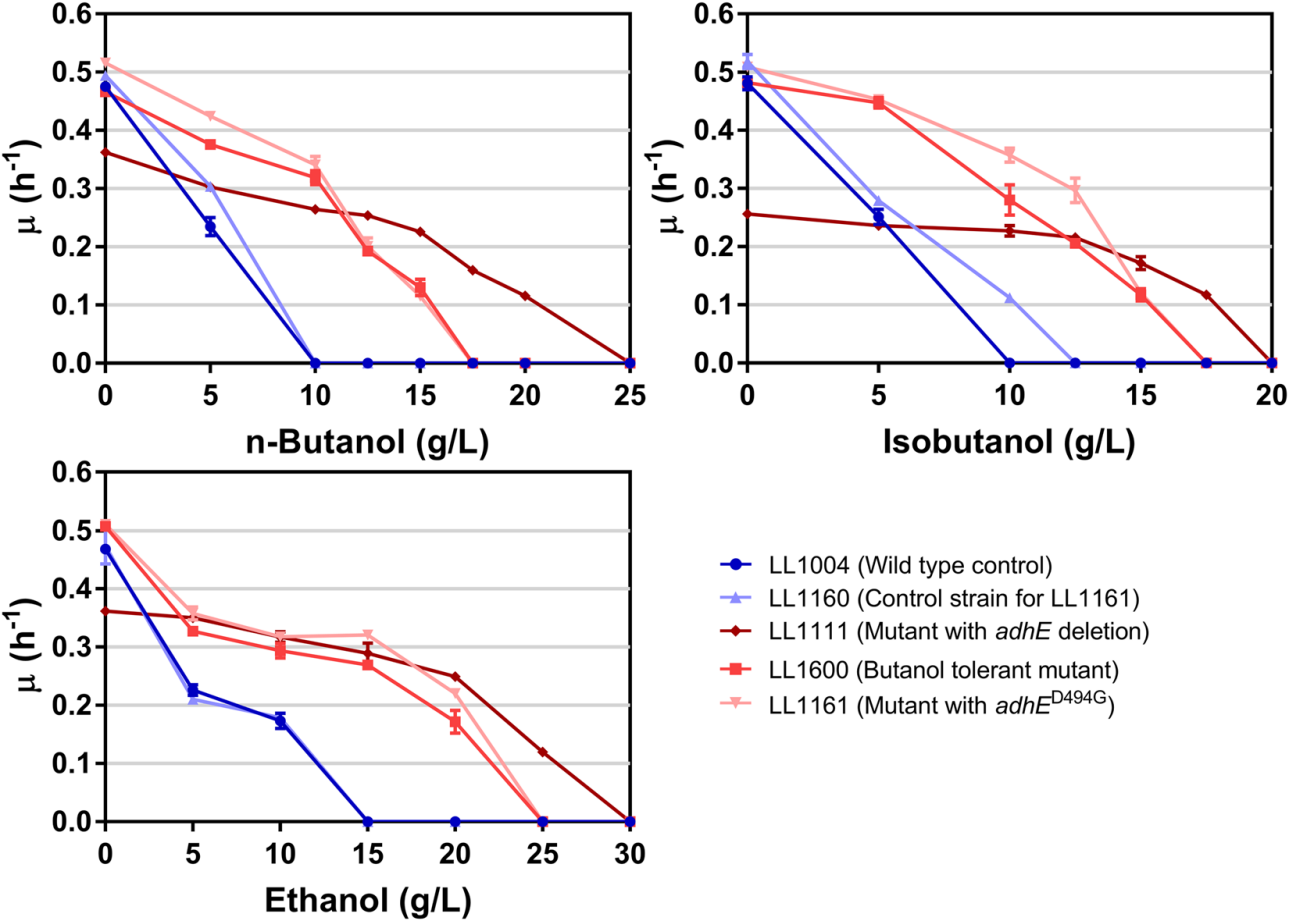
Tolerance of the reconstructed *C*. *thermocellum* mutant to ethanol, n-butanol and isobutanol. Growth rate was measured in MTC-5 medium at 55 °C. Maximum specific growth rate is plotted against the concentration of the alcohol. The data represents the average of three biological replications. Error bars represent one standard deviation. Strains colored in shades of blue are wild type for the *adhE* gene. Strains colored in shades of red have mutations or deletion in the *adhE* gene. The dark blue circle represents the wild type strain LL1004; the light blue triangle (up-direction) represents the control strain, LL1160; The dark red diamond represents the *adhE* deletion strain, LL1111; the red square represents the selected n-butanol tolerant strain, LL1600; the light red triangle (down-direction) represents the strain with the mutant *adhE* (D494G mutation, strain LL1161).

For the strain with the mutant *adhE* gene (LL1161), n-butanol tolerance was similar to that of the n-butanol-adapted strain (LL1600, Table 3) (Fig. 3). Since we know that mutations in the AdhE protein have been shown to increase ethanol tolerance^26,32^, we hypothesized that this mutation might increase tolerance to other primary alcohols. This is, in fact, what we found when we tested tolerance to isobutanol (Fig. 3).

### Effect of AdhE mutations

Ethanol production in *C*. *thermocellum* involves successive reduction of acetyl-CoA and acetaldehyde with electrons provided by NADH (i.e., the ALDH and ADH reactions). These two reactions are both catalyzed by the bifunctional AdhE enzyme. Previously we have shown that as ethanol concentrations increase, the NADH/NAD^+^ ratio also increases^20^. In the case of *added* ethanol, this may be due to reverse flux through the ADH and ALDH reactions, however even with *produced* ethanol, when net flux is in the direction of ethanol formation, the NADH/NAD^+^ ratio may need to increase to maintain a negative Gibbs free energy change for the ALDH and ADH reactions. High NADH/NAD^+^ ratios have been shown to inhibit the GAPDH reaction^33,34^ which inhibits the carbon flux of glycolysis in *C*. *thermocellum*^20^. The increased NADPH-linked activity associated with the AdhE^D494G^ mutation provides a potential explanation for this. In this mutant, reverse flux through the ADH reaction can affect both the NADH/NAD^+^ ratio and the NADPH/NADP^+^ ratio. Changes in the NADPH/NAD^+^ ratio, however, will not affect the GAPDH reaction, which is strictly NADH-linked in *C*. *thermocellum* (Table S1).

To apply the same explanation for n-butanol and isobutanol tolerance, we need to demonstrate that AdhE can also use butyryl-CoA/isobutyryl-CoA and butyraldehyde/isobutyraldehyde as substrates. Both the wild type AdhE and mutant AdhE were cloned and purified in *E*. *coli* and their activities were measured (Table 2). From these results, we confirmed that AdhE has the capacity to catalyze both butyryl-CoA conversion to butyraldehyde and isobutyryl-CoA conversion to isobutyraldehyde. In addition to lending support for our hypothesis that isobutanol and n-butanol inhibit *C*. *thermocellum* metabolism by inhibiting the GAPDH reaction, results from enzyme assays provide insight into the native pathway for isobutanol production in this organism, the final steps of which were previously unknown^23^.

**Table 2:** Kinetic parameters of purified *C*. *thermocellum* wild type and D494G mutant AdhE proteins.

Substrate	Cofactor	AdhE			AdhE D494G		
		K_m_ (µM)	V_max_ (U/mg)^a^	k_cat_/K_m_ (M^−1^s^−1^)	K_m_ (µM)	V_max_ (U/mg)	k_cat_/K_m_ (M^−1^s^−1^)
**Forward Direction**							
acetyl-CoA	NADH	38.3 ± 1.5^b^	1.84 ± 0.13	7.69E + 04	45.8 ± 2.1	1.51 ± 0.11	5.28E + 04
acetaldehyde		4658 ± 594	1.21 ± 0.09	4.16E + 02	5136 ± 485	1.04 ± 0.08	3.24E + 02
butyryl-CoA		75.7 ± 3.7	3.08 ± 0.21	6.51E + 04	84.5 ± 5.5	2.88 ± 0.19	5.45E + 04
butyraldehyde		4152 ± 485	6.14 ± 0.42	2.37E + 03	4351 ± 407	5.37 ± 0.34	1.98E + 03
Iso-butytyl-CoA		54.5 ± 2.8	4.18 ± 0.23	1.23E + 05	65.5 ± 4.8	3.78 ± 0.19	9.24E + 04
Iso-butyradehyde		3578 ± 485	5.84 ± 0.32	2.61E + 03	3897 ± 414	5.78 ± 0.34	2.37E + 03
acetaldehyde	NADPH	ND^c^			4754 ± 214	1.45 ± 0.21	4.88E + 02
butyraldehyde					3457 ± 248	8.51 ± 0.41	3.94E + 03
Iso-butyradehyde					3789 ± 234	6.25 ± 0.21	2.64E + 03
**Reverse Direction**							
**Substrate**	**Cofactor**	**AdhE**			**AdhE D494G**		
		**K**_**m**_ **(mM)**	**V**_**max**_ **(U/mg)**	**k**_**cat**_ **/K**_**m**_ **(M** ^**−1**^ **s** ^1^ **)**	**K**_**m**_ **(mM)**	**V**_**max**_ **(U/mg)**	**k**_**cat**_ **/K**_**m**_ **(M** ^**−1**^ **s** ^**−1**^ **)**
ethanol	**NAD** ^**+**^	59.06 ± 6.01	3.79 ± 0.60	1.03E + 02	57.21 ± 5.01	3.14 ± 0.42	8.78E + 01
n-butanol		62.21 ± 5.54	2.98 ± 0.54	7.67E + 01	58.32 ± 6.75	2.18 ± 0.23	5.98E + 01
Isobutanol		65.7 ± 6.12	2.58 ± 0.46	6.28E + 01	61.40 ± 4.54	2.94 ± 0.32	7.67E + 01
ethanol	NADP^+^	ND			48.51 ± 6.32	3.52 ± 0.63	1.16E + 02
n-butanol					53.14 ± 4.58	2.59 ± 0.68	7.80E + 01
Isobutanol					51.15 ± 3.65	2.77 ± 0.59	8.67E + 01

^a^1U = 1 µmol/min.

^b^The data represents the average of three individual rounds of assays.

^c^ND = not detected.

To further confirm our hypothesis that reverse flux through AdhE is the mechanism for inhibition by primary alcohols, we tested the tolerance of a strain with an *adhE* deletion (strain LL1111). As shown in Fig. 3, the *adhE* deletion strain shows tolerance to all three primary alcohols and demonstrated the highest level of tolerance among the strains tested.

## Conclusions

After isolating a butanol tolerant mutant of *C*. *thermocellum*, we discovered that the strain had acquired a D494G mutation in the *adhE* gene. We showed that this mutation is sufficient to recapitulate the n-butanol tolerance phenotype. We further showed that this is applicable to other primary alcohols including ethanol and isobutanol. To show that reverse flux through AdhE is the mechanism of inhibition, we confirmed that it has NADH-linked activity with both isobutyryl-CoA and butyryl-CoA. Furthermore, deletion of the *adhE* gene increases tolerance for all three primary alcohols tested. The mechanism found in this study can be widely applied to other organisms.

## Methods

### Bacterial strains, media and cultivation

Strains used in this study are listed in Table 3. All chemicals were reagent grade and obtained from Sigma-Aldrich (St. Louis, MO) or Fisher Scientific (Pittsburgh, PA) unless indicated otherwise. CTFUD rich medium^35^ and MTC-5 defined medium^36^ were used for routine strain maintenance and strain evolution.

**Table 3:** Strains used in this work.

Organism	Strain number	Description	Accession number	Source or reference
*C*. *thermocellum*	LL1004	wild type strain DSM 1313	CP002416	DSMZ
	LL1599	n-butanol tolerant mutant isolate 1	SRP163075	this work
	LL1600	n-butanol tolerant mutant isolate 2	SRP163076	this work
	LL1601	n-butanol tolerant mutant isolate 3	SRP163072	this work
	LL1603	n-butanol tolerant mutant isolate 4	SRP163070	this work
	LL1604	n-butanol tolerant mutant isolate 5	SRP163069	this work
	LL1605	n-butanol tolerant mutant isolate 6	SRP163071	this work
	LL1111	LL1004 *Δhpt ΔadhE ldh*(S161R)	SRX744221	^21^
	LL1160	LL1111 *adhE ldh*(S161R)	SRA273168	^31^
	LL1161	LL1111 *adhE*^D494G^ *ldh*(S161R)	SRA273169	^31^
	AG929	DSM1313 *Δhpt ΔClo1313_0478*	SRP097241	^41^
	LL1636	*AG929 ΔClo1313_0853*		this work
*E*. *coli*	BL21(DE3)	T7 Express *lysY/lq* Used for heterologous protein expression		New England Biolabs
	DH5α	DH5α Used for plasmid screening and propagation		New England Biolabs
		BL21(DE3) overexpressing *C*. *thermocellum adhE*		^39^
		BL21(DE3) overexpressing *C*. *thermocellum adhE*^*D494G*^		^39^
		BL21(DE3) overexpressing *C*. *thermocellum gapdh*		^20^

### Strain evolution

Serum bottles cultures were incubated at 55 °C and shaken at 180 rpm. Serum bottles were purged with N_2_ and sealed with butyl rubber stoppers. In batch cultures, pH was regulated with 40 mM MOPS buffer. Chemostat bioreactor fermentations were carried out in 0.5 L (100 ml working volume) bioreactors (NDS Technologies Ins, Vineland NJ) in modified MTC-5 medium without MOPS buffer and with 2 g/L urea as the nitrogen source, with the temperature maintained at 55 °C and stirred at 150 rpm. The dilution rate was set to 0.04 h^−1^. The pH was controlled at 7.0 with a Mettler-Toledo pH probe (Columbus, OH) by the addition of 8 N KOH. The bioreactor was inoculated with 5 mL fresh culture grown on 5 g/L cellobiose in MTC-5. The headspace of the bioreactor was flushed with N2 gas prior to inoculation. The feed bottle was continuously purged with N_2_ gas to maintain anaerobic conditions. Appropriate amounts of n-butanol were added to the feed bottle. To avoid selecting an inducible mutation (as opposed to a constitutive mutation), the n-butanol concentration was reduced to 0 g/L twice during the selection process. 16 s rRNA gene sequences of cell pellets from the fermentation were used to verify culture purity. The adapted culture was first grown on CTFUD agar plates and then 12 isolates were selected and inoculated into liquid CTFUD medium.

### Tolerance test

Tolerance was measured in a COY (Ann Arbor, MI) anaerobic chamber (85% N_2_, 10% CO_2_, and 5% H_2_). 200 µl cultures were grown in 96-well pre-sterilized polystyrene plates in an anaerobic chamber (85% N_2_, 10% CO_2_, and 5% H_2_). Absorbance measurements at 600 nm (OD_600_) were taken every 10 minutes for 36 hours using a BioTek plate reader (BioTek Instruments Inc., Winooski, VT). Growth rates were determined based on the slope of blank-subtracted, log-transformed absorbance data in the range of 0.1 to 1. Averages were based on at least three independent biological replicates.

### Protein purification

For expression and purification of proteins in *E*. *coli*, cell preparation and cell free extract were prepared as described previously^37^. Cells were grown aerobically in TB medium at 37 °C with a stirring speed of 225 rpm. When the OD_600_ reached 0.6, 4 mM rhamnose was added to induce expression of the target gene. Cells were then grown aerobically for 4 h before harvesting by centrifugation. Cell pellets were washed with buffer (50 mM Tris-HCl, pH 7.5 and 0.5 mM DTT) and stored at −80 °C.

The cell pellet was resuspended in lysis buffer (1X BugBuster reagent (EMD Millipore, Darmstadt, Germany) with 0.2 mM dithiothreitol). The cells were lysed with Ready-Lyse lysozyme (Epicentre, Madison, WI, USA), and DNase I (New England Biolabs, Ipswich, MA, USA) was added to reduce the viscosity. After incubation for 30 min at room temperature, the resulting solution was centrifuged at 10,000 X g for 5 min. The supernatant was used as cell free extract for enzyme assays or protein purification.

All purification steps were performed at room temperature as described previously^38^. His-tag affinity spin columns (His SpinTrap; GE Healthcare BioSciences, Pittsburgh, PA, USA) were used to purify the protein. The column was first equilibrated with binding buffer (50 mM sodium phosphate, 500 mM NaCl, 20 mM imidazole, pH 7.5). Cell free extracts (in 50 mM sodium phosphate, 500 mM NaCl, 20 mM imidazole, pH 7.5) were applied to the column, and then the column was washed twice with wash buffer (50 mM sodium phosphate, 500 mM NaCl, 50 mM imidazole, 20% ethanol, pH 7.5). The His-tagged protein was eluted with elution buffer (50 mM sodium phosphate, 500 mM NaCl, 500 mM imidazole, pH 7.5).

### Enzyme assays

Enzyme assays were performed in a COY (Ann Arbor, MI) anaerobic chamber (85% N_2_, 10% CO_2_, and 5% H_2_, <5 ppm O_2_). ALDH and ADH activities were measured as described previously^39^. The reaction mix contained: 50 mM pH 7.0 Tris-HCl buffer (The pH was adjusted at 55 °C to avoid changes in pH with changes in temperature), 2 mM MgCl_2_, 0.5 mM DTT, 5 µM FeSO_4_, 0.3 mM NADH or NADPH. For ALDH activity, six different substrate concentrations between 10 and 500 µM of acetyl-CoA, butyryl-CoA or isobutyryl-CoA were used as substrates to start the reaction. For ADH activity, six different substrate concentrations between 0.25 to 20 mM acetaldehyde, butyraldehyde or isobutyraldehyde were used. The consumption of NADH or NADPH was followed spectrophotometrically at 340 nm (molar extinction coefficient ε of NADH/NADPH = 6.22 mM^−1^cm^−1^) in a BioTek PowerWave XS plate reader (BioTek Instruments Inc., Winooski, VT, USA).

The activity of the glyceraldehyde-3-phosphate dehydrogenase enzyme (GAPDH EC 1.2.1.12) was measured at 55 °C as previously described^40^. The standard assay (200 µl working volume) contained 50 mM Tris-HCl pH 7.0, 10 mM sodium arsenate, 10 mM glyceraldehyde-3-phosphate, and 0.5 mM NAD^+^. To avoid thermal destruction of glyceraldehyde-3-phosphate, this substrate was added to the mixture immediately before starting the enzyme reaction. The formation NADH or NADPH were followed by photometric observation at 340 nm (ε = 6.2 mM^−1^ cm^−1^) in a BioTek PowerWave XS plate reader (BioTek Instruments Inc., Winooski, VT, USA).

The protein concentration was determined using the Bradford protein reagent with bovine serum albumin as the standard (BioRad, Hercules, CA).

## Supplementary information

Table S1 : Kinetic parameters of C. thermocellum GAPDH enzyme.

Figure S1: Growth of C. thermocellum in thepresence of different concentrations of n-butanol from 0 to 8 g/L.
