# Supplementary figures and images for: A mutation in the AdhE alcohol dehydrogenase of Clostridium thermocellum increases tolerance to several primary alcohols, including isobutanol, n-butanol and ethanol
Source: Sci Rep. 2019 Feb 11;9:1736. doi: 10.1038/s41598-018-37979-5 (PMC6370804; doi:10.1038/s41598-018-37979-5)

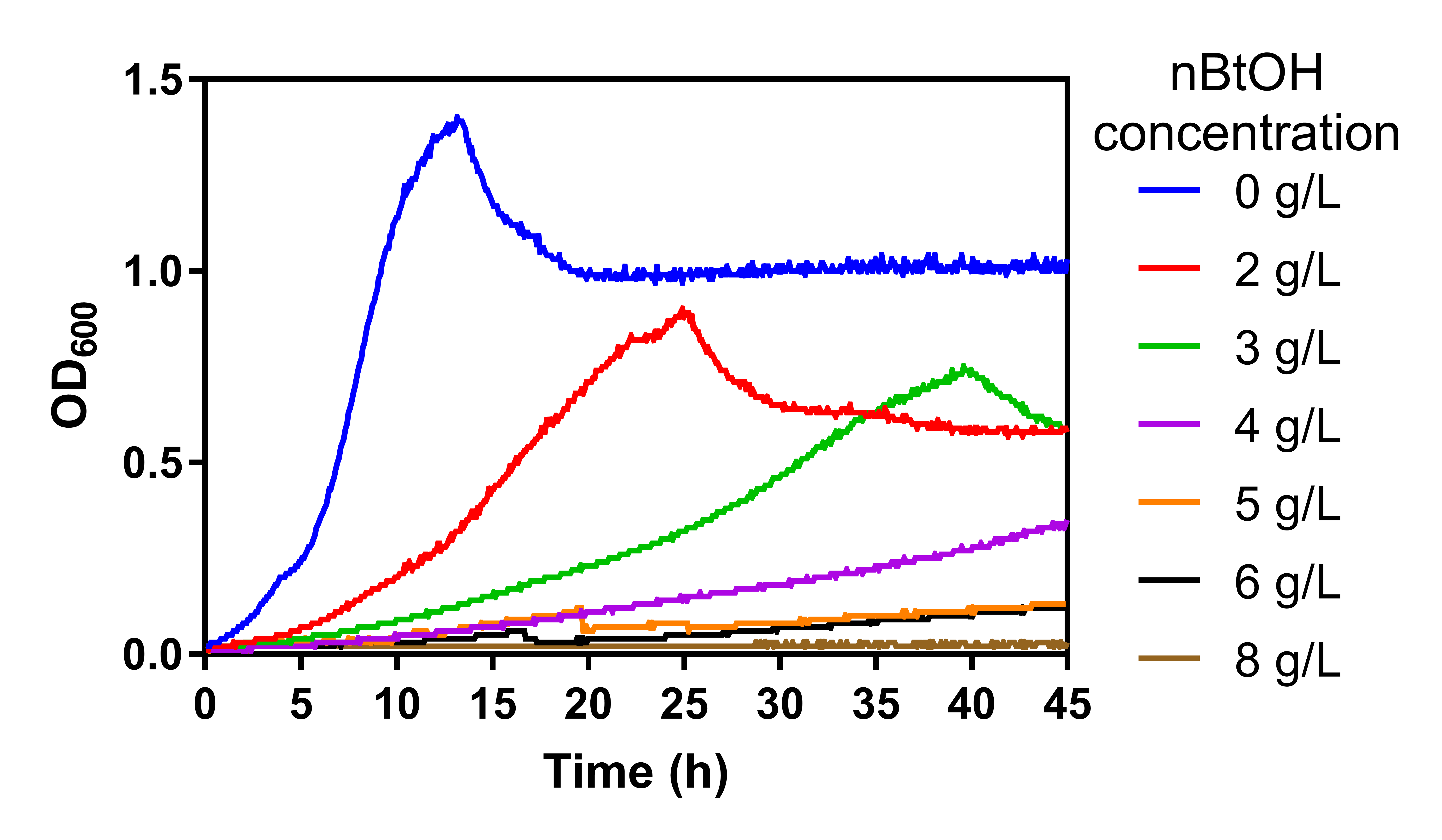

Supplement: Supplementary file 2 — Figure S1: Growth of C. thermocellum in thepresence of different concentrations of n-butanol from 0 to 8 g/L. [file 41598_2018_37979_MOESM2_ESM.tif]
